# Effects of different amylose to amylopectin ratios on rumen fermentation and development in fattening lambs

**DOI:** 10.5713/ajas.17.0833

**Published:** 2018-04-12

**Authors:** Fangfang Zhao, Wen Ren, Aizhong Zhang, Ning Jiang, Wen Liu, Faming Wang

**Affiliations:** 1 College of Animal Science and Veterinary Medicine, Heilongjiang Bayi Agricultural University, Daqing 163319, China; 2 Laboratory of Metabolic Manipulation of Herbivorous Animal Nutrition, College of Animal Science and Technology, Yangzhou University, Yangzhou 225009, China; 3 DSM China Animal Nutrition Research Centre Co. Ltd, Bazhou 165700, China

**Keywords:** Lambs, Rumen Fermentation Parameters, Rumen Morphology, Rumen Weight and Volume, Starch

## Abstract

**Objective:**

The objective of this experiment was to examine the effects of different amylose/amylopectin ratios on rumen fermentation and development of fattening lambs.

**Methods:**

Forty-eight 7-day-old male Small-tailed Han sheep×Northeast fine wool sheep were randomly assigned to four treatments of dietary amylose/amylopectin ratios (0.12, 0.23, 0.24, and 0.48 in tapioca starch, corn starch, wheat starch and pea starch diets, respectively). Three lambs from each treatment were slaughtered at 21, 35, 56, and 77 days of age to determine the rumen fermentation and development.

**Results:**

Compared with tapioca starch diet, the pea starch diet significantly increased the concentration of ammonia nitrogen in the ruminal fluid of lambs but significantly decreased the bacterial protein content. At 56 and 77 d, the rumen propionate concentration tended to be greatest in the tapioca starch group than in other groups. The rumen butyrate concentration was the greatest in lambs fed on pea starch compared with those fed on other starch diets. Furthermore, the pea starch diet significantly stimulated rumen development by increasing the papillae height, width and surface area in the rumen ventral or dorsal locations in lambs. However, different amylose/amylopectin ratios diets did not significantly affect the feed intake, body weight, average daily gain, the relative weight and capacity of the rumen in lambs with increasing length of trial periods.

**Conclusion:**

Lambs early supplemented with a high amylose/amylopectin ratio diet had favourable morphological development of rumen epithelium, which was not conducive to bacterial protein synthesis.

## INTRODUCTION

In ruminants, the early transition from simple gastric digestion to functional ruminal digestion is essential for their health and growth [1]. The rumen of a newborn ruminant will undergo several anatomical and physiological changes during this transition. These changes, and thus ruminal development, are essentially affected by the solid feed intake and its composition [1,2].

Carbohydrates are the main energy source for ruminants, and feeding diets that differ in carbohydrate composition may result in different patterns of rumen fermentation, subsequently resulting in different quantities and proportions of volatile fatty acid (VFA). These differences, in turn, may differentially affect rumen development. Therefore, the rational application of starch as a primary energy substance of carbohydrates substantially impacts the growth and health of ruminants. Native starch is mostly composed of two distinct types of glucose polymers: amylose (liner glucose polymers) and amylopectin (highly branched glucose polymers). The amylose to amylopectin ratio varies in different starch sources, and a high amylose to amylopectin ratio is negatively correlated with starch digestion [3]. Offner et al [4] has also noted that there is a large variability in starch ruminal degradation among various starch sources. Starch digestion rates influence the amount of starch that is degraded in the rumen, which may affect the profile of VFA in the rumen and thus ruminal development. Therefore, studies on the effects of the dietary amylose to amylopectin ratio on early development of young ruminants are great importance to improve their health. Ren et al [5] reported that higher amylose to amylopectin ratio diets could promote higher concentrations of plasma cholesterol, lactate dehydrogenase and growth hormone and could up-regulate the gene expression profiles of glucose transporters in weaning lambs. To our knowledge, scientific evaluating the effects of different amylose to amylopectin ratios on rumen fermentation and development in lambs is quite limited. Due to the different fermentation characteristics of various starch sources, it is hypothesized that starch with different amylose/amylopectin ratios will influence rumen fermentation and development of fattening lambs. Here, we fed diets containing different amylose/amylopectin ratios (tapioca starch group, 0.12; corn starch, 0.23; wheat starch, 0.24; pea starch, 0.48) for fattening lambs during preweaning, weaning and early postweaning periods and investigated rumen fermentation and rumen development, and the objective was to find a suitable amylose/amylopectin diet for lambs from the perspective of the rumen.

## MATERIALS AND METHODS

### Animal care

All experimental animals received care according to the Guide for the Care and Use of Laboratory Animals by the Chinese Academy of Science.

### Diets and feeding

Forty-eight single-born hybrid male lambs (Small-tailed Han sheep×Northeast fine wool sheep) averaging 3.81 kg of birth weight (standard error = 1.2 kg) were obtained from the Yinlang sheep farm (Daqing, China). The lambs were separated from their mothers at seven days of age and placed in individual pens bedded with wood shavings. Then, the lambs were randomly assigned to four treatments of dietary amylose to amylopectin ratios (12 lambs per treatment). All lambs were fed 1.4 L/d of lamb milk replacement from a nipple bottle from 7 to 21 days of age, 1.2 L/d from 22 to 35 days of age, and 0.8 L/d until weaning at 56 days of age. Lambs received milk replacement four times daily from 7 to 49 days of age and twice daily from 50 to 55 days of age. The commercial milk replacement contained 21% crude protein (CP) and 12% fat at 150 g/L (dry matter [DM] basis). Different starch diets and alfalfa hay were supplied in separate buckets and feeds were provided *ad libitum* (15% refusals) and twice daily at 08:00 and 20:00. Lambs received concentrate/roughage ratio was 85/15 (DM basis). All lambs were provided free access to water from a bowl drinker in their own pen. During the trial period, the maximum, minimum and mean temperatures registered in the pens were 28.0°C, 14.5°C, and 20.2°C, respectively.

The experimental concentrates contained 50.2% starch, 29.0% soybean meal, 14.8% corn protein meal, 1.6% soybean oil, 0.8% limestone, 1.2% CaHPO_4_, 0.4% salt, and 2.0% premix (DM basis). The chemical composition of the dietary treatments was 19.8% CP, 14.2 MJ/kg metabolizable energy, 0.8% Ca, and 0.6% P. The premix provided a per kg of pellet diet as follows, Fe 25 mg, Zn 40 mg, Cu 8 mg, Mn 40 mg, I 0.3 mg, Se 0.2 mg, Co 0.1 mg, vitamin A 940 IU, vitamin E 20 IU. The ratios of amylose/amylopectin in diets were as follows: tapioca starch group (TS, amylose/amylopectin = 0.12), corn starch group (CS, amylose/amylopectin = 0.23), wheat starch group (WS, amylose/amylopectin = 0.24), and pea starch group (PS, amylose/amylopectin = 0.48). The experimental diets were formulated to meet the nutrition requirements for fattening lambs at a medium growth speed according to the NRC [6]. All starch sources were purchased from a company (Shanghai Dragon and Food Co., Ltd, Shanghai, China). Alfalfa hay was the only forage source. The chemical composition of alfalfa hay was 92.5% for DM, 16.7% for CP, 1.4% for Ca, 0.9% for P. The chopped length of the alfalfa hay was approximately 3 cm.

### Growth performance

The amounts of the diets were recorded daily. All lambs were weighed on the morning every week after birth. The average daily gain (ADG) was calculated based on the weights.

### Sample collection and measurements

The sequence of slaughter was properly balanced across treatments. Three lambs from each group were randomly selected, weighed and slaughtered by captive bolt stunning at 11:00 to 12:00 (after 3 to 4 h from morning feeding) on 21, 35, 56, and 77 days of age. The abdominal cavity was immediately opened. The rumen, reticulum, omasum and abomasum were dissected and individually weighed with their contents. Next, they were emptied, rinsed repeatedly with cool water, drained, and reweighed. The volume of each stomach compartment was accessed using the method developed by Stobo et al [7]. The parameters of rumen weight as a percentage of body weight, rumen weight as a percentage of all stomach compartments, and the rumen relative capacity as a percentage of all stomach compartments were calculated. At d 21, 35, 56, and 77 of age, ruminal contents were collected approximately 3 to 4 h post-feeding and were filtered through four layers of cheesecloth, then the rumen fluid pH was immediately determined using a pH electrode (Model PHS-3C, Shanghai Precision & Scientific Instrument CO., LTD, Shanghai, China). A sample of rumen fluid was stored at −20°C to determine the ammonia nitrogen and bacterial protein contents. The ammonia nitrogen content was assessed using the colorimetric method described by Chaney and Marbach [8]. The bacterial protein content was evaluated based on the method by Cotta et al [9] and Broderick and Craig [10]. After centrifugation of 10 mL rumen fluid (10,000 g, at 4°C for 15 min), a 5 mL aliquot supernatant was acidified with 1 mL of 25% *ortho*-phosphoric acid solution and stored at −20°C for analysis of the VFAs. The VFA concentration was analysed using high-performance liquid chromatography (LC-15C, SHIMADZU, Kyoto, Japan) and 0.25 mM sulphuric acid as the mobile phase as described by Suárez et al [11]. The tested volume was 1 μL and the flow rate was 1 mL/min. Detection was completed using a refractive index detector. Calibration and quantification were performed using an external standard solution.

For the morphometrical analysis, one tissue specimen (approximately 1 cm^2^) was taken from the saccus ruminis dorsalis (dorsal location) and one specimen was taken from the saccus ruminis ventralis cranial of the pilae ruminis (ventral location). The tissue samples were fixed in 10% formaldehyde solution. After rinsing with water, the samples were dehydrated in a graded series of ethanol solution (50%, 70%, 80%, 90%, and 100%), cleared twice with benzene, and saturated and embedded in paraffin according to the method by Wang et al [12]. Six sections were taken from each sample, and each slice was prepared at a distance of at least 100 μm from the nearest slice. Paraffin sections of 7 μm in thickness were made and stained with haematoxylin and eosin, then observed and measured under a light microscope. The morphometric analysis was performed at 40× magnification (Olympus, Tokyo, Japan) using an image analysis software program (Mshot Digital Imaging System, Guangzhou, China). The surfaces of the papillae were determined as height×width. All morphometric measurements were performed by the same person, who had no prior knowledge of the correspondence between samples and treatments.

### Chemical analyses

Samples of the lambs’ diets were analysed for DM and CP content according to the Association of Official Analytical Chemists (AOAC [13]). Starch content was determined according to the procedure of Hall [14]. The diets were analyzed to determine the contents of amylose and amylopectin using the procedure described by Englyst et al [15]. The calcium and phosphorus contents were measured using inductively coupled plasma emission spectroscopy with an Atom Scan 25 Plasma Spectroscopy (Thermo Jarrell Ash Corp., Grand Junction, CO, USA) after acid digestion.

### Statistical analysis

The data were analyzed as a completely randomized design, in a 4×4 factorial arrangement, with diets and length of the experimental age (i.e., d 21, 35, 56, and 77 of age at slaughter) as main factors. The general linear model procedure of SAS Version 9.2 (SAS Institute Inc., Cary, NC, USA) was used for the analysis, and the model was as follows:

$$\text{Y}=\mu +{\text{diet}}_{\text{i}}+{\text{age}}_{\text{j}}+{\left(\text{diet}\times \text{age}\right)}_{\text{ij}}+{\text{e}}_{\text{ij}}$$

Where Y is the dependent variable, μ is the overall mean, diet_i_ is the effect of different amylose to amylopectin ratios (TS, CS, WS, and PS); age_j_ is the effect of age (age at slaugther) j; j is d 21, 35, 56, and 77 of age; (diet×age)_ij_ is the effect of the interaction between diet and age; e_ij_ is the random error term. The term diet×age was removed from the model when the effect of interaction was not significant. The Tukey test was used to identify differences (p<0.05) between means.

## RESULTS

### Growth performance

All lambs remained healthy throughout the experiment. The measurments of feed intake, dry matter intake (DMI), body weight and ADG of lambs fed different amylose/amylopectin diets at different days of age are showed in Table 1. Feed intake, DMI, body weight and ADG were affected by age (p<0.001) but not by diet and they were greater as the lambs aged.

### Rumen fermentation

The effects of different ratios of dietary amylose/amylopectin on ruminal pH, ammonia nitrogen contents and bacterial protein contents are shown in Table 2. Ruminal pH was not affected by diet and age. Ammonia nitrogen contents were affected by diet (p<0.01) and age (p<0.001). Lambs fed the PS diet had higher ruminal ammonia nitrogen content than lambs fed the TS, CS, or WS diets (p<0.01). Ruminal ammonia nitrogen content was greatest (p<0.001) in lambs at d 77 of age followed in those at d 35 and 56 of age and then in those at d 21 of age. Bacterial protein contents also were affected by diet (p<0.01) and age (p<0.001). TS-fed lambs had higher bacterial protein content than lambs fed the PS, CS, or WS diets (p<0.01). Bacterial protein content was greatest (p<0.001) in lambs at d 77 of age followed in those at d 35 and 56 of age and then in those at d 21 of age.

The results of the concentrations of short-chain fatty acids are presented in Table 3. Total volatile fatty acid (TVFA) concentrations were affected by age (p<0.001) but not by diet and decreased at 56 days of age compared with 35 days of age. Concentrations of acetate, valerate, isobutyrate and branch chain VFA were affected by age (p<0.001, p<0.01, p<0.001, and p<0.001, respectively) but not by diet (p>0.05). At d 35, 56, and 77 days of age, the propionate concentrations of lambs fed the PS diet was lower than those of lambs fed the TS diet (p<0.001). Butyrate concentrations also were affected by diet (p<0.001) and age (p<0.001). Moreover, an interaction between diet and age also was observed (p<0.01). At d 35, 56, and 77 of age, the butyrate concentrations of lambs fed the PS diet was higher than those of lambs fed the TS, WS, and CS diets (p<0.001). The isovalerate concentration was not affected by diet and age.

### Rumen development

The measurements of the relative weight and the relative volume of the rumen tissue according to treatment are shown in Table 4. Rumen weight as % of body weight and rumen weight as % of all compartments of stomach were not affected by diet but by age (p<0.001). Rumen weight as % of body weight and rumen weight as % of all compartments of stomach were greatest (p<0.001) in lambs at d 56 and 77 of age followed in those at d 35 of age and then in those at d 21 of age. The relative capacity of the rumen as a percentage of all stomach compartments also was not affected by diet but by age (p< 0.001). The relative volume of the rumen was greater as the lambs aged.

The rumen morphometric measurements are summarized in Table 5. Five parameters, including the height, width, density and surface area of the rumen papillae, and the muscular layer thickness, were used to determine the effects of different ratios of dietary amylose/amylopectin on the ruminal morphology of fattening lambs at 21, 35, 56, and 77 days of age. Papillae height was affected by diet and lambs fed the PS diet had higher papillae height than lambs fed the TS diet both in the rumen dorsal (p<0.01) and rumen ventral locations (p< 0.001). Both diet (p<0.01) and age (p<0.001) effects were presented in papillae width in the dorsal location and ventral location. In the dorsal location of the rumen, lambs fed the PS diet had a significantly increased papillae width compared with lambs fed the TS, CS, and WS diets, respectively. In both locations, the papillae width increased with increasing length of the trial period. Surface area of papillae was affected by diet (p<0.01, p<0.001) and age (p<0.001) both in the rumen dorsal and rumen ventral locations. In the rumen dorsal location, PS-fed lambs had a significantly increased papillae surface area compared with lambs fed TS, CS, and WS diets. However, no significant difference was observed between PS group and WS group in the rumen ventral location. Muscular layer thickness and papillae density were not affected by diet but by age (p< 0.001). Muscular layer thickness increased with increasing length of the experimental period, whereas papillae density decreased with increasing length of the trial period.

## DISCUSSION

In rearing young ruminants, intake of milk replacer is an important factor influencing DMI [16]. In the present study, the DMI of the concentrate diets varied between 239.26 and 245.05 g/d (Table 1), but no significant effect was observed among four treatments. That may be due to the control of milk replacer amount among four treatments in this experiment and that is conducive to compare the difference caused by amylose/amylopectin ratios in concentrate. The increased feed intake, DMI, body weight and ADG with age was an exception and that related to the improvement of gastrointestinal capacity and growth characteristics.

Early supplementation with a starter promotes rumen development by stimulating the fermentation process [17]. An increase in starter consumption by ruminants as they aged could enhance the capacity of their gastrointestinal tract to accommodate and ferment solid feed [2,18]. However, early supplementation with different starters may produce distinct effects. In the current study, there were some significant differences between four different treatments in the rumen fermentation products and morphometrical measurements. Interestingly, the rumen pH was unaffected by the source of starch in the current experiment. This outcome was somewhat unexpected because it is well known that the rumen degradability of pea starch is much lower than that of corn starch, wheat starch and tapioca starch [4]. Furthermore, the degradation rate of dietary starch has a major impact on rumen pH [19]. Though the rumen pH did not significantly differ depending on the amylose/amylopectin ratio of the starch source, diets with different amylose/amylopectin ratios may result in a variation in the change rate of rumen pH in lambs because amylose-rich starch has a slower degradation rate, which could lead to a slower rate of organic acid production. In the present study, lambs fed the PS diet showed higher ammonia nitrogen content (18.66 mg/100 mL; Table 2). That may be attributed to the rumen degradability of starch sources. It has been reported that the accumulation of ammonia nitrogen was negatively correlated with the degradation rate of carbohydrates [20]. The result was also found in our trial. The increased accumulation of ammonia nitrogen may have been caused by the decrease in bacterial protein synthesis. Correspondingly, the results of the current study showed that the PS diet significantly decreased the rumen bacterial protein content. The decreased bacterial protein content in lambs fed the PS diet may have also been related to the poor synchronous utilization of starch and protein. Ammonia nitrogen contents (14.96 to 20.06 mg/100 mL) and bacterial protein contents (0.54 to 1.57 mg/mL) increased with increasing length of the experimental period may be attributed to greater protein intake and its degradation by ruminal microbes.

Ruminal fermentation is established by solid feed intake in the neonate. The process produces VFAs that subsequently stimulate ruminal development [17]. Different carbohydrates may result in different fermentation patterns [21]. Our study showed that ruminal TVFA was unaffected by different dietary amylose to amylopectin ratios among the four treatment groups. The similar ruminal TVFA concentrations may have been caused by similar dietary intakes, whereas the decrease in ruminal TVFA content of lambs at 56 days of age compared to those at 35 days of age in all lambs may be attributed to an increased capacity of the ruminal epithelium to absorb VFA [22]. The period effect on TVFA concentration (Table 2) may be ascribed to greater solid feed and probably better fermentation of organic matter by ruminal microbes. Amylopectin-rich starches have a greater ruminal degradability compared to amylose-rich starches, which could lead to more available starch for fermentation by rumen microorganisms [23]. This is likely the cause of the greater ruminal propionate content of the lambs at d 35, 56, and 77 of age in the TS group. Moreover, the results from the current study showed that the greater ruminal butyrate concentration of lambs fed the PS (amylose-rich starch) diet at 35, 56, and 77 days of age compared with those fed the TS (amylopectin-rich starch) diet. The results for the ruminal butyrate concentrations were similar to those of a previous study [24], which found increased butyrate concentrations in the caeca and colons in growing pigs fed a raw pea starch diet (amylose-rich starch). That finding was also supported by another study [25], which reported that potato starch (amylose-rich starch) effectively increased the butyrate concentration in the human colon. In the current study, there were no significant differences in the short-chain fatty acids concentrations of lambs both in the CS and WS groups. This result may be explained by the similar amylose to amylopectin ratios of the starch sources in these two groups.

The mass, volume and morphology of the rumen change rapidly when lambs are transitioned from their neonatal reliance on milk-supplied nutrients to solid-feed supplied nutrients (from cereal grains and hay), namely, the transition from glucose to VFAs as the main energy source. These great changes in lambs are derived from the physical stimulation of solid feeds and the chemical stimulation of solid feed fermentation products. Previous studies have shown that physical stimulation by solid feed in the rumen could account for measurable increases in rumen mass, volume and muscularization [26]. In the present study, there was no significant difference in the relative weights and relative capacities of the stomach compartments in lambs regardless of treatment. This result may be attributed to the similar physical stimuli caused by similar consumption of solid feed. However, the relative weight and the relative volume of the rumen were greater as the lambs aged. This rise indicated greater physical stimuli as a consequence of an increased consumption of solid feed.

Greater ruminal papillae height, width and papillae density in lambs may be ascribed by better chemical stimuli because of fermentation of solid feed. Ruminal physical development is characterized by increases in rumen weight and volume and by the growth of the papillae. Favourable development of the rumen papillae is crucial for nutrient utilization [27]. Most previous studies have demonstrated that use of ruminally inert materials to simulate the physical stimulus of feed in the rumen results in no significant development of the papillae [28]. Conversely, the infusion of sodium butyrate results in marked papillary development in calves [28] and lambs [29]. These results suggest that the presence of a physical stimulus alone does not promote the development of papillae. Therefore, for the normal development of the ruminal epithelium, there should be viable rumen fermentation that produces short-chain fatty acids in the ruminal lumen in order to trigger papillary growth [30]. It has been reported that the stimulatory effects of different VFAs on papillary development are not equal, with butyrate being the most stimulatory [28]. The underlying mechanism for this response may involve the regulation of insulin-like growth factor-I production by butyrate, a process which in turn increases the size of the papillae [31]. In addition, a previous study [32] demonstrated that butyrate and glucose are readily oxidized by isolated lamb rumen epithelial cells, which can provide energy for ruminal epithelium development. Thus, the PS diet significantly (high amylose/amylopectin) stimulated papillary development by increasing the height, width and surface area of the papillae in the rumen ventral or dorsal locations may be by the consequence of higher ruminal butyrate concentration.

## Figures and Tables

**Table 1:** Feed intake, body weight and ADG of different diets amylose/amylopectin at different days of age in lambs

Variable	Diet				SEM	p-value1)			Age				SEM
		
	TS	CS	WS	PS		D	A	D×A	d 21	d 35	d 56	d 77	
Feed intake (g/d)	258.21	254.26	258.76	272.64	7.59	NS	***	NS	41.44^d^	106.54^c^	309.14^b^	586.75^a^	7.59
Dry matter intake (g/d)	242.97	239.26	243.49	245.05	7.04	NS	***	NS	38.57^d^	98.97^c^	287.49^b^	545.743^a^	7.04
Body weight (kg)	13.34	13.97	13.85	14.58	0.65	NS	***	NS	8.37^d^	10.67^c^	15.46^b^	21.26^a^	0.65
ADG (g/d)	179.37	180.39	180.37	181.74	4.15	NS	***	NS	166.50^c^	171.37^c^	184.54^b^	199.47^a^	4.15

TS, tapioca starch group; CS, corn starch group; WS, wheat starch group; PS, pea starch group; SEM, standard error of the mean.

1) D, diet; A, age; D×A, interaction.

*** p<0.001, NS, not significant.

a,b,c,d Within a row, different superscripts indicate significant difference between means (p<0.05).

**Table 2:** Average ruminal pH, ammonia nitrogen contents and bacterial protein contents in lambs fed different amylose/amylopectin diets at different days of age

Variable	Diet				SEM	p-value1)			Age				SEM
		
	TS	CS	WS	PS		D	A	D×A	d 21	d 35	d 56	d 77	
Rumen pH	6.77	6.82	6.86	6.73	0.07	NS	NS	NS	6.80	6.88	6.79	6.70	0.07
Ammonia nitrogen (mg/100 mL)	17.53^b^	17.64^b^	17.49^b^	18.66^a^	0.34	**	***	NS	14.96^c^	18.14^b^	18.16^b^	20.06^a^	0.34
Bacterial protein (mg/mL)	1.39^a^	1.25^b^	1.28^b^	1.17^b^	0.04	**	***	NS	0.54^c^	1.37^b^	1.44^b^	1.57^a^	0.04

TS, tapioca starch group; CS, corn starch group; WS, wheat starch group; PS, pea starch group; SEM, standard error of the mean.

1) D, diet; A, age; D×A, interaction.

** p<0.01;

*** p<0.001. NS, not significant.

a,b,c Within a row, different superscripts indicate significant difference between means (p<0.05).

**Table 3:** Effects of different dietary amylose to amylopectin ratios on rumen fluid concentrations of short-fatty acids at different days of age

Variable	Diet				SEM	p-value1)			Age				SEM
		
	TS	CS	WS	PS		D	A	D×A	d 21	d 35	d 56	d 77	
Total VFA (mmol/L)	44.98	43.13	43.56	44.46	1.10	NS	***	NS	34.69^d^	45.51^b^	40.36^c^	55.57^a^	1.10
Acetate	27.41	26.10	26.41	27.31	0.89	NS	***	NS	24.24^c^	27.86^b^	23.67^c^	31.47^a^	0.89
Propionate						***	***	**					
d 21	5.86	5.34	5.73	5.63	0.25	-	-	-	-	-	-	-	-
d 35	10.48^a^	10.78^a^	10.83^a^	8.38^b^	0.55	-	-	-	-	-	-	-	-
d 56	9.65^a^	8.52^b^	8.02^b^	7.61^b^	0.29	-	-	-	-	-	-	-	-
d 77	17.12^a^	15.15^b^	15.08^b^	14.85^b^	0.45	-	-	-	-	-	-	-	-
Butyrate						***	***	^*^					
d 21	1.72	1.9	2.04	1.73	0.37	-	-	-	-	-	-	-	-
d 35	2.75^c^	3.10^b^	3.21^b^	3.75^a^	0.06	-	-	-	-	-	-	-	-
d 56	2.81^c^	3.37^b^	3.52^b^	4.65^a^	0.12	-	-	-	-	-	-	-	-
d 77	2.72^b^	2.72^b^	2.99^b^	4.21^a^	0.30	-	-	-	-	-	-	-	-
Valerate	1.36	1.41	1.36	1.57	0.12	NS	**	NS	0.97^b^	1.47^a^	1.58^a^	1.68^a^	0.12
Isobutyrate	1.61	1.67	1.65	1.66	0.09	NS	***	NS	0.83^d^	1.90^b^	1.40^c^	2.45^a^	0.09
Isovalerate	1.33	1.22	1.29	1.23	0.13	NS	NS	NS	1.17	1.46	1.18	1.27	0.13
Branch chain VFA	2.93	2.89	2.94	2.88	0.16	NS	***	NS	2.00^c^	2.86^b^	3.07^b^	3.72^a^	0.16

TS, tapioca starch group; CS, corn starch group; WS, wheat starch group; PS, pea starch group; SEM, standard error of the mean; VFA, volatile fatty acid.

1) D, diet; A, age; D×A, interaction.

** p<0.01;

*** p<0.001. NS, not significant.

a,b,c,d Within a row, different superscripts indicate significant difference between means (p<0.05), When there are significant difference of the interaction D and A, it is showed in 4×4 line-column.

**Table 4:** Rumen measurements of lambs under different dietary amylose to amylopectin ratios at different days of age

Variable	Diet				SEM	p-value1)			Age				SEM
		
	TS	CS	WS	PS		D	A	D×A	d 21	d 35	d 56	d 77	
Rumen weight as % of body weight	2.02	2.16	1.87	1.88	0.10	NS	***	NS	0.10^c^	1.62^b^	2.54^a^	2.78^a^	0.10
Rumen weight as % of weight of all compartments of stomach	62.31	64.15	61.38	61.71	1.34	NS	***	NS	48.20^c^	61.61^b^	69.24^a^	70.50^a^	1.34
Rumen volume as % of volume of all compartments of stomach	75.00	73.77	74.95	74.91	0.62	NS	***	NS	62.07^d^	71.74^c^	80.30^b^	84.52^a^	0.62

TS, tapioca starch group; CS, corn starch group; WS, wheat starch group; PS, pea starch group; SEM, standard error of the mean.

1) D, diet; A, age; D×A, interaction.

** p<0.01;

*** p<0.001. NS, not significant.

a,b,c,d Within a row, different superscripts indicate significant difference between means (p<0.05).

**Table 5:** Effects of dietary amylose to amylopectin ratios on rumen morphology in lambs at different days of age

Variable	Diet				SEM	p-value1)			Age				SEM
		
	TS	CS	WS	PS		D	A	D×A	d 21	d 35	d 56	d 77	
Rumen dorsal location													
Papillae height (μm)	1,014.42^b^	1,028.93^ab^	1,029.65^ab^	1,060.54^a^	13.57	**	***	NS	588.46^d^	833.03^c^	1,292.76^b^	1,419.29^a^	13.57
Papillae width (μm)	316.36^b^	310.75^b^	310.65^b^	337.51^a^	6.33	**	***	NS	285.50^c^	277.69^c^	342.99^b^	369.10^a^	6.33
Surface area of papillae (mm^2^)	0.33^b^	0.33^b^	0.33^b^	0.37^a^	0.01	***	***	NS	0.17^d^	0.23^c^	0.44^b^	0.52^a^	0.01
Muscular layer thickness (μm)	441.86	439.38	444.08	439.22	5.10	NS	***	NS	394.99^c^	418.21^b^	410.38^b^	540.97^a^	5.10
Papillae density (number/cm^2^)	101	98	101	96	2.06	NS	***	NS	115^a^	110^a^	91^b^	80^c^	2.06
Rumen ventral location													
Papillae height (μm)						***	***	^*^					
d 21	581.00	584.44	589.67	598.72	11.45	-	-	-	-	-	-	-	-
d 35	800.50	839.11	835.72	856.78	24.14	-	-	-	-	-	-	-	-
d 56	1,279.06	1,272.44	1,295.72	1,323.83	34.02	-	-	-	-	-	-	-	-
d 77	1,397.11	1,419.72	1,397.5	1,462.83	41.03	-	-	-	-	-	-	-	-
Papillae width (μm)	302.42^b^	317.51^ab^	311.72^ab^	322.44^a^	5.09	**	***	NS	245.60^d^	282.11^c^	340.60^b^	385.79^a^	5.09
Surface area of papillae (mm^2^)	0.34^b^	0.35^b^	0.36^ab^	0.39^a^	0.01	**	***	NS	0.15^d^	0.25^c^	0.46^b^	0.59^a^	0.01
Muscular layer thickness (μm)	452.08	467.32	452.42	454.08	5.95	NS	***	NS	394.57^d^	422.21^c^	472.36^b^	536.76^a^	5.95
Papillae density (number/cm^2^)	96	95	95	98	1.21	NS	***	NS	115^a^	103^b^	87^c^	79^d^	1.21

TS, tapioca starch group; CS, corn starch group; WS, wheat starch group; PS, pea starch group; SEM, standard error of the mean.

1) D, diet; A, age; D×A, interaction.

** p<0.01;

*** p<0.001. NS, not significant.

a,b,c,d Within a row, different superscripts indicate significant difference between means (p<0.05), When there are significant difference of the interaction D and A, it is showed in 4×4 line-column.
